# Glioblastoma single-cell microRaman analysis under stress treatments

**DOI:** 10.1038/s41598-018-26356-x

**Published:** 2018-05-22

**Authors:** Maria Ricci, Francesco Ragonese, Beatrice Gironi, Marco Paolantoni, Assunta Morresi, Loredana Latterini, Bernard Fioretti, Paola Sassi

**Affiliations:** 1 Institute of Materials and Environmental Chemistry, Research Centre for Natural Sciences of the Hungarian Academy of Sciences, Magyar tudósok körútja 2, 1117 Budapest, Hungary; 2 Dipartimento di Medicina Sperimentale, Università di Perugia, Via Gambuli 1, 06132 Perugia, Italy; 3 Dipartimento di Chimica Biologia e Biotecnologie, Università di Perugia, Via Elce di sotto 8, 06123 Perugia, Italy; 4 Centro di Eccellenza sui Materiali Innovativi Nanostrutturati (CEMIN), Università di Perugia, Via Elce di Sotto 8, 06123 Perugia, Italy

## Abstract

Glioblastoma multiforme (GBM) is the most frequent malignant brain tumor characterized by highly heterogeneous subpopulations. In order to reveal the heterogeneous cell response, single cell analysis is an essential requirement. In this study, optical microscopy and Raman microspectroscopy were used to follow the stress response of U251 single cells adherent on a silicon substrate. Cultured cells on silicon substrate were treated with hydrogen peroxide to promote apoptosis. Under these conditions expected changes occurred after a few hours and were revealed by the reduction of cytochrome c, lipid, nucleic acid and protein Raman signals: this ensured the possibility to analyse U251 cell line as grown on Si substrate, and to monitor the response of single cells to stress conditions. As a consequence, we used microRaman to monitor the effects induced by nutrient depletion: a fast change of Raman spectra showed two different sub-populations of sensible and resistant U251 cells. Furthermore, spectral variations after DMSO addition were associated to volume changes and confirmed by morphological analysis. Thus, our results highlight the sensitivity of Raman microspectroscopy to detect rapid variations of macromolecule concentration due to oxidative stress and/or cell volume changes at the single cell level.

## Introduction

Glioblastoma multiforme (GBM) is the most frequent malignant astroglial-derived tumour in adults. The average survival rate from the time of diagnosis is less than twelve months, and even in the least aggressive forms, GBM causes most patients to die within two year time frame^1,2^. Numerous studies have focused on gaining a better understanding of different molecular mechanisms exploited by invading GBM tumour cells^3–5^ and in recent years there has been much interest in the use of optical tools for cancer diagnostics because of their ability to detect biochemical changes occurring at the early stages of tumorigenesis^6^. Aside from being one of the most invasive and deadly human malignancies, GBM is a example model of a heterogeneous cancer^7,8^. Its heterogeneity as well as the capacity to counteract against an hostile microenvironment, cause the conventional and targeted treatments to fail a long-term remission^9,10^. In order to reveal heterogeneous cell responses, analysis at the single cell level is an essential requirement^11^. In the last few years, there has been a rapid expansion of high throughput single cell analysis, also due to an increasing use of microfluidic devices for the total analysis of single cells^12,13^. At present, for single cell detection, fluorescence techniques such as Fluorescence Resonance Energy Transfer, Quantitative Time-Lapse Fluorescence Microscopy and Super-Resolution Fluorescence methods, remain the most common methods used^14,15^. These techniques share the limitation of their dependency on the use of probes, which can affect the cell balance and homeostasis. In this respect, it is noted that Raman microspectroscopy has been recognized as a powerful technique not solely for the single cell analysis but also for the non-invasive investigation of living cells^16,17^. Indeed, this technique allows the assessment of the overall molecular composition of the sample without requiring cell fixation, staining or lysis. Therefore, it can represent an efficient, non-destructive tool for the analysis of single living cells and the characterization of their dynamic biochemical processes^18–20^.

In this work, the adhesion of GBM cells to silicon substrates was evaluated and Raman microspectroscopy was used to identify molecular markers for a label-free monitoring of the dynamic stress events in single cells. The biochemical variations were induced by addition of an apoptotic inducer such as, hydrogen peroxide (H_2_O_2_), nutrient depletion or by addition of dimethyl sulfoxide (DMSO). External stimulus, like a change in nutrient composition or a chemical treatment, is potentially harmful, since it can induce a cell response including various morphological and biochemical modifications, or even cell death^20^. Cell swelling represents a “marker” that occurs in response to a diversity of cellular stress, such as physical damage, metabolic stress (nutrient depletion and hypoxia) and chemical stress (es. Methylmercury)^21,22^. Several mechanisms are involved in cell swelling such as Cl^−^/HCO_3_^−^ and Na^+^/H^+^ exchange transport systems or ions (sodium, potassium and chloride) channels activity^21^. Generally, the uptake of Na^+^ leads to increased intracellular osmolarity and swelling. Regulatory Volume Decrease (RVD) phase based on efflux of organic osmolyte such as taurin or salt, like KCl, follows the swelling phase to restore the normal volume size^23,24^. RVD involves activation of conductive K^+^ and Cl^−^ channels, allowing for the escape of KCl and osmotically obligated water^21^.

## Results

The cell adhesion is the result of a dynamic process related to specific interactions between the substrate surface and cell ligands and is highly depended on the cell and substrate types^25^. For this reason, it was necessary to assess the adhesion of the U251 cells to the Si substrate.

**Figure 1:**
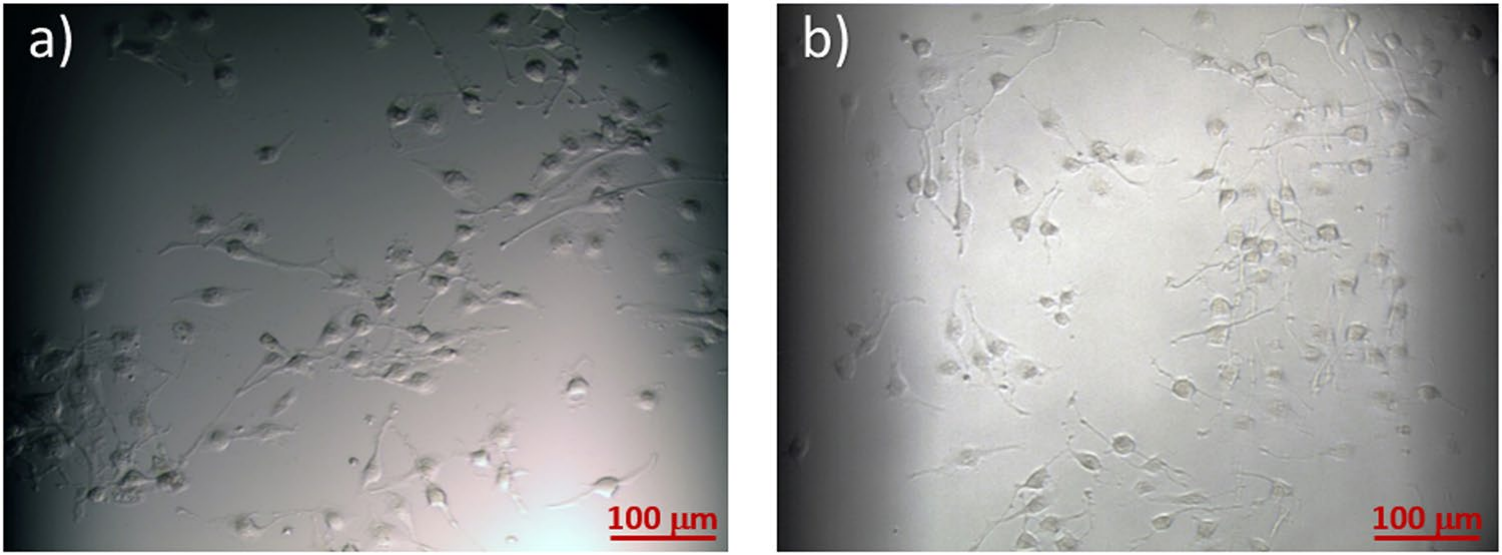
Optical images of U251 cells. 10x magnification images of U251 cells grown on a Si substrate **(a)** and on a plastic petri dish (**b**).

**Figure 2:**
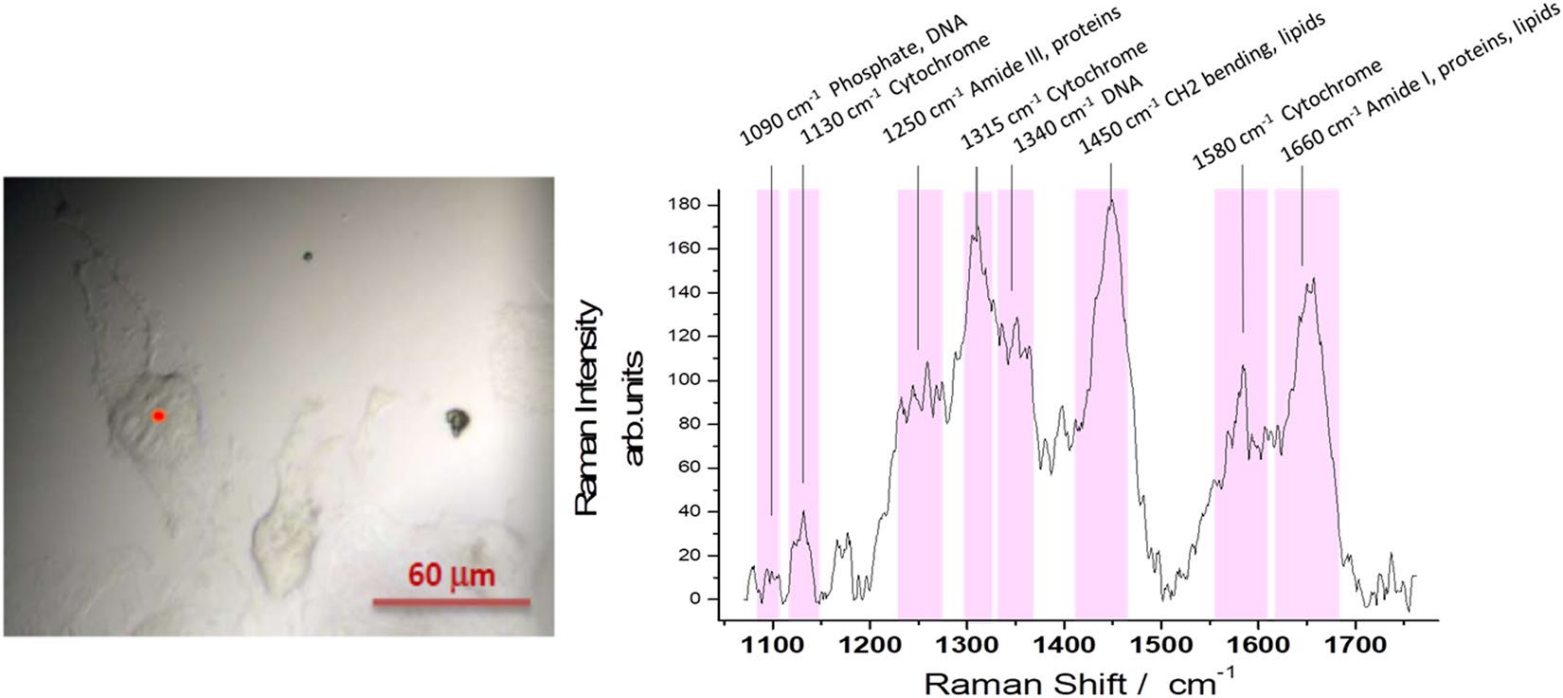
Microspectroscopy of U251 cells (**a**) 50x magnification image of U251 cells. (**b**) Raman spectrum of the cell point indicated with the red spot in (**a**) with peak assignments^16,20,34,35^.

The compatibility of the substrate for cell adherence and the normal growth was verified by comparing the bright field images of cells grown on Si (Fig. 1a) and on plastic typically used for cell cultures (Fig. 1b). In both cases, the cells grow flat stretched to the substrate surface indicating favourable conditions. Figure 2 shows Raman spectrum of U251 cells grown on silicon with the vibrational assignment of the fingerprint region (Fig. 2b). Despite that silicon spectral contributions hide the region below 1060 cm^−1^, it is possible to follow the variation of lipid, DNA and protein as well as cytochrome c (cyt c) bands.

### Spectroscopic markers of apoptosis

The evolution of both morphology and Raman signals was followed on cells treated for 4 hours with 300 μM H_2_O_2_, which is known to be able to induce apoptosis^26,27^.

**Figure 3:**
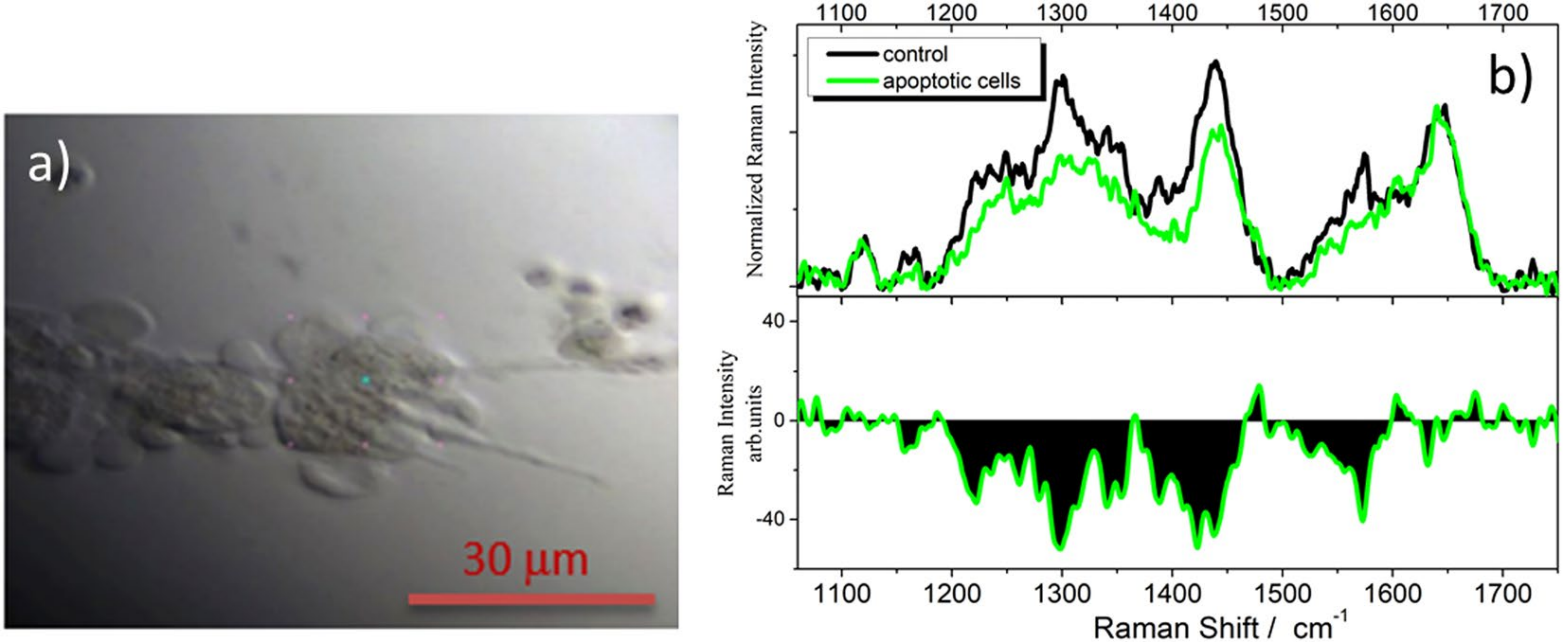
Microspectroscopy of H_2_O_2_-treated cells. (**a**) 50x magnification image of U251 cells treated for 4 hours with 300 μM H_2_0_2_ and analysed by Raman microspectroscopy. (**b**) Comparison between average Raman spectra of apoptotic (green line) and non-apoptotic (black line) cells; on lower panel the difference spectrum obtained by subtracting the control to the spectrum of apoptotic cells is shown.

Membrane blebbing was evident after H_2_O_2_ treatment (Fig. 3a). In order to identify the variations associated with the apoptotic process in U251 cells, the Raman signals from the central region of the cells (in correspondence of the nucleus) were detected. Figure 3b displays the average Raman spectrum of different U251 cells grown on silicon and treated with hydrogen peroxide. By comparing this spectrum with the non-apoptotic control, it is possible to note that the apoptotic cells the cyt c, lipid, DNA and protein signals have a reduced Raman intensity.

At the beginning of the apoptotic process, cyt c can act as a trigger of the activation of the caspase cascade, resulting in the disassembly of proteins^28^. In particular, Okada *et al*.^29^ observed the decrease of the Raman signal intensity at 1580 cm^−1^ associated to the translocation of cyt c from mitochondria to the cytosol as a first response to induced apoptosis; they also show that actomycine D unmodify the redox status of cyt c during mitochondrial release^28^. In our case, when cells are treated with H_2_O_2_, a decrease of cyt c bands is rather due to the ferrous to ferric oxidation of heme site. In fact, the two intense Raman bands at 1315 and 1580 cm^−1^ of the reduced form turn to be very weak when the oxidized state of the heme site prevails. These Raman signals are assigned to the skeletal vibrations of the protoporphyrin ring of heme unit and are recognized to be good probes of the oxidation state of iron in cyt c^30,31^.

The decrease of the lipid band at 1450 cm^−1^ also shown in Fig. 3b (upper and lower panels), is reasonably related with the loss of lipid molecules due to apoptotic membrane blebbing formation on the cell surface^32^ (Fig. 3a). Finally, the intensity loss of protein and nucleic acid bands in the 1200–1350 cm^−1^ region, resulting in a strong negative contribution to the difference spectrum of Fig. 3b, is consistent with the action of H_2_O_2_^26^ and with the activity of proteases and DNases (deoxyribonuclease) during the apoptotic process^33,34^.

### Glucose depletion promotes heterogeneous response of U251 cell line

The effects of a condition of nutrient depletion was studied and compared with glioblastoma cells treated with H_2_O_2_. During nutrient depletion, cells are able to adapt their metabolism to starvation imposed by decreased extracellular nutrients, such as glucose, or by decreased intracellular metabolite concentrations. In U251 cell line, glucose deprivation increases necrotic cell death^35^ that is often associated to membrane depolarization, oxidative stress and cell swelling^21,36^.

**Figure 4:**
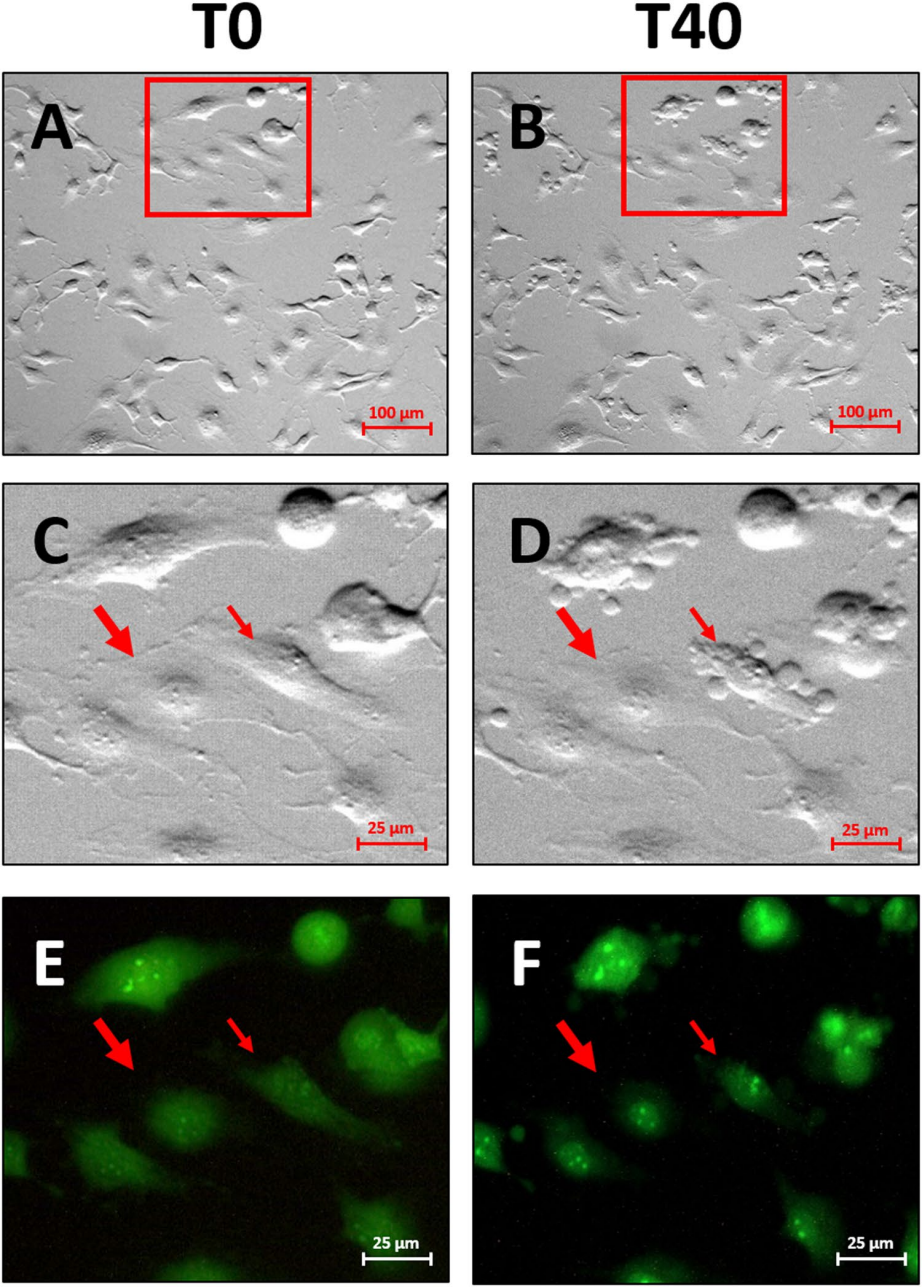
U251 cells during nutrient depletion. Image of U251 before (**A** and **C**) and after (**B** and **D**) 40 min with PBS at different magnification rate. Notable the co-presence of two subpopulation in the same culture area (tick arrow: nutrients depletion resistant cell; thin arrow: nutrients depletion sensible cell). Detail of same cell stained with Acridine Orange before (**E**) and after (**F**) 40 min with PBS. No lysosome formation is visible after 40 min of exposure to PBS.

Two principal cellular subpopulations have been observed during nutrients depletion condition in agreement with heterogeneous metabolic phenotype: nutrients depletion sensible (NDSC) and nutrients depletion resistant cells (NDRC). Based on microscopy analysis, NDSC population was characterized by significant changes in cell morphology during starvation with evident cell swelling and membrane blebbing, whereas in NDRC no evident morphological changes were observed (Fig. 4A–D). All these phenomena were not related to an autophagic behaviour, since no autophagosome formation was observed with acridine orange assay (Fig. 4E,F). In contrast, Trypan blue assay displays an increase of necrotic cell percentage after two hours in PBS (data not shown); this suggests that membrane blebbing in NDSC may be associated to an early necrosis process. MicroRaman analysis was performed on these two subpopulations.

#### Nutrients depletion sensible cells (NDSC)

**Figure 5:**
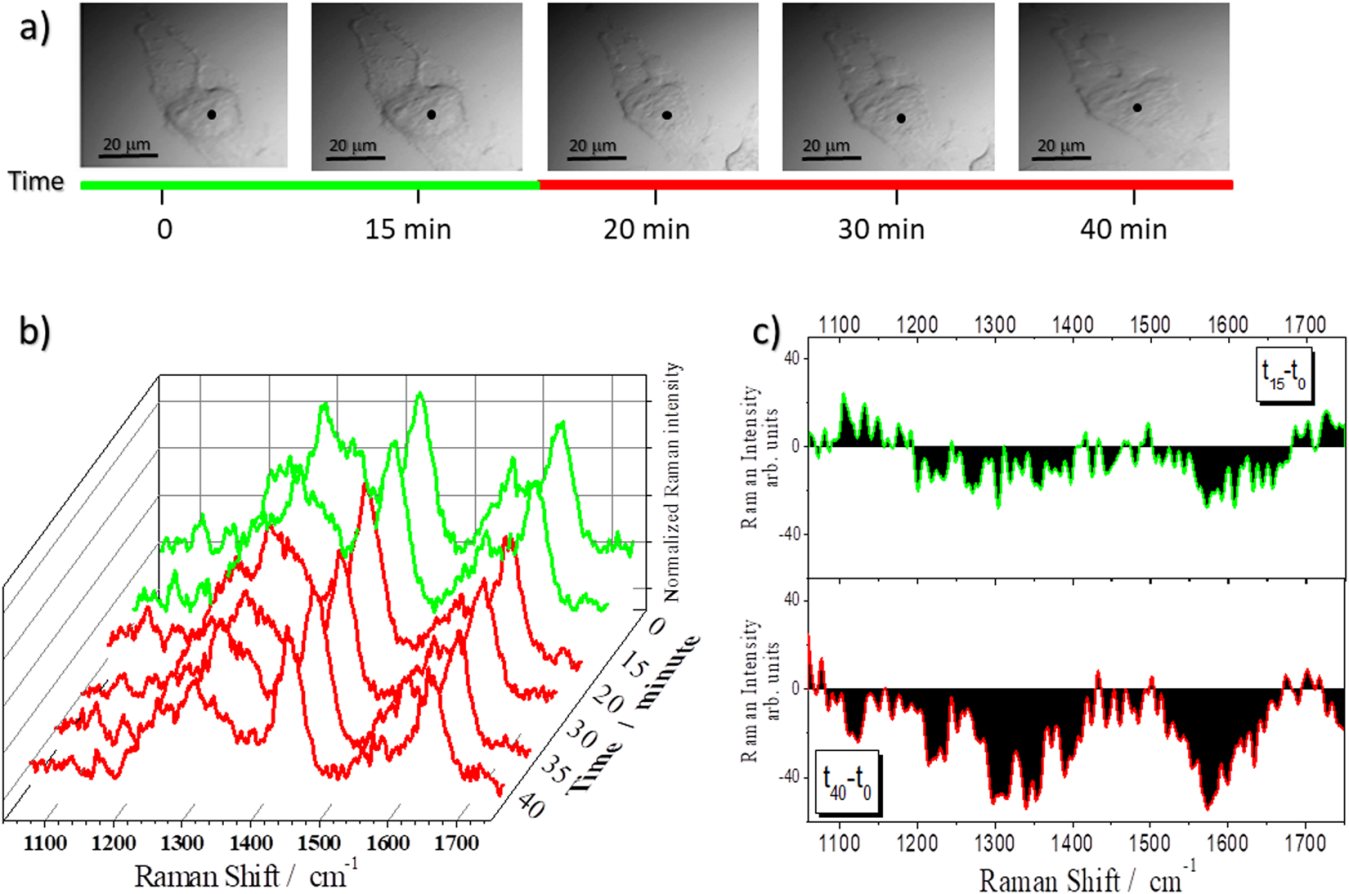
Monitoring the effects of starvation: NDSC. (**a**) 50x magnification images of a U251 cell monitored as a function of time. (**b**) Raman spectra collected in the cell point indicated by the black spot in (**a**) at different times. The colour of the spectra is intended to indicate the cell status: green for viable cells, red for stressed cells. (**c**) Differences between treated spectra at 15 (t_15_) and 40 (t_40_) minutes and control (t_0_).

Figure 5 displays the evolution of the Raman spectra acquired every 5 minutes upon application of nutrients depletion condition. NDSC were classified based on morphological changes (Fig. 5a). The main spectral changes occur after 20 minutes from the removal of the medium (Fig. 5b, 20 min): they involve the intensity loss of the cyt c bands at 1314 cm^−1^ and 1580 cm^−1^, and the decrease of the nucleic acid signal at 1337 cm^−1^ in the same period of time. On the other hand, no significant changes occur within lipid and protein features. These spectroscopic results are parallel with morphological changes of the cell (Fig. 5a, 20 min). After 30 minutes, the pronounced decrease of cyt c and nucleic acid signals at 1337 cm^−1^ and 1090 cm^−1^ is observed (Fig. 5b, 30 min). After 35 minutes, it is also possible to note that the protein band at 1250 cm^−1^ decreases in intensity (Fig. 5b, 35 min). The spectral variations are also shown in Fig. 5c: subtle differences at 15 minutes after removal of the medium are observed in the 1200–1600 cm^−1^ region; at 40 minutes these differences are much stronger, particularly at 1580 cm^−1^ and in the 1300–1350 cm^−1^ range. The variations are monitored together with morphological changes typical of cell swelling. Whereas until 15 minutes from the beginning of the experiment the cell retains its shape (Fig. 5a, 15 min), there is a clear morphology variation after 20 minutes (Fig. 5b, 20 min) which is clearly visible on the cell protrusions. At the late time, the change of the cell shape can be associated with the formation of protuberances on the surface (Fig. 5a, 20–40 min).

At the end of the experiment, all the spectral components show a reduced intensity apart from the lipid band at 1450 cm^−1^ (Fig. 5b, 40 min and lower panel of Fig. 5c): this is also the main difference with the spectrum of the H_2_O_2_ treated cells (see Figs 3b and 5c).

#### Nutrients depletion resistant cells (NDRC)

**Figure 6:**
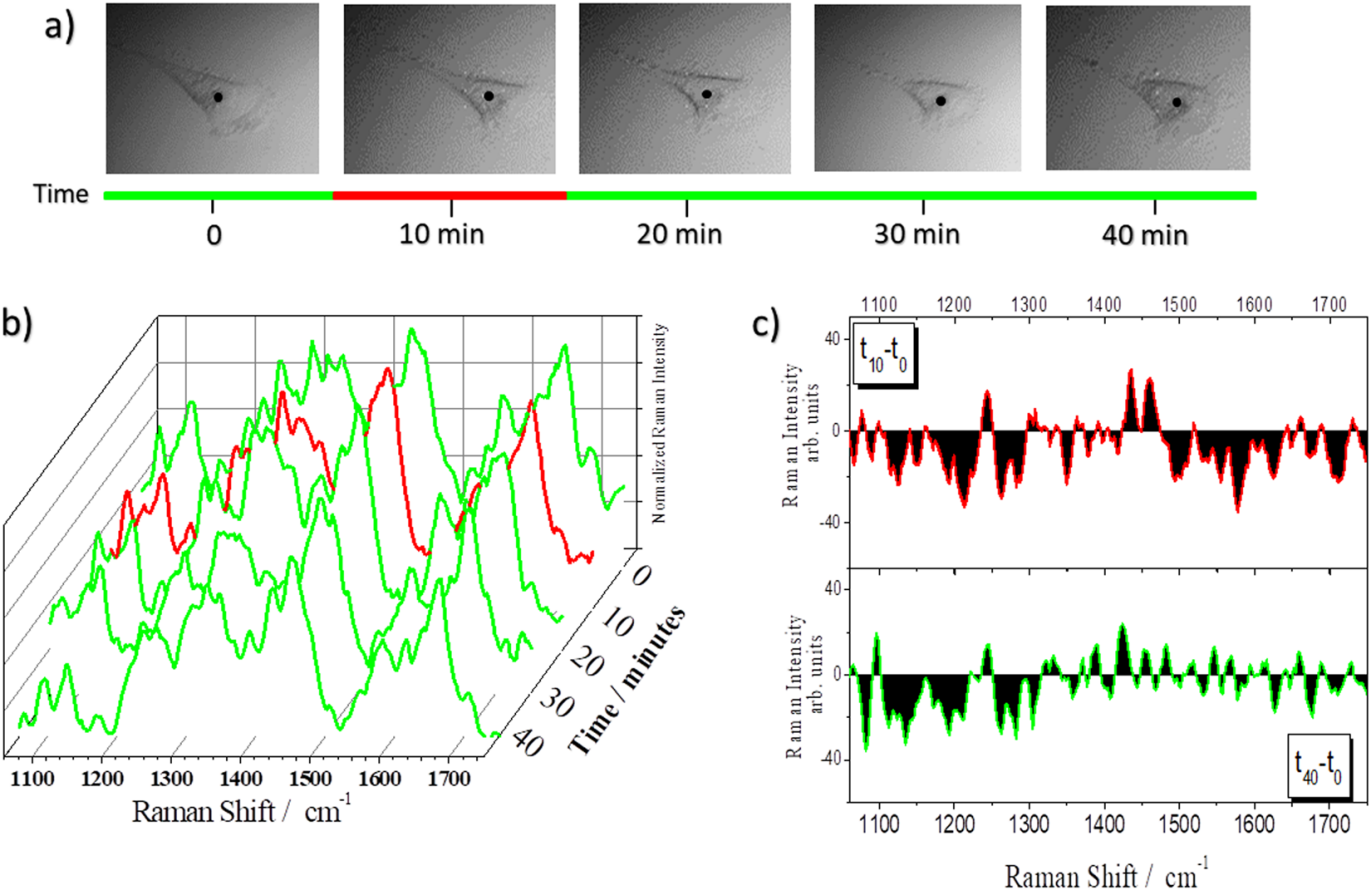
Monitoring the effects of starvation: NDRC. (**a**) 50x magnification images of a U251 cell monitored as a function of time. (**b**) Raman spectra collected in the cell point indicated by the black spot in (**a**) at different time points. The colour of the spectra is intended to indicate the cell status: green cell viability, red cell stress. (**c**) Differences between treated spectra at 10 (t_10_) and 40 (t_40_) minutes and control (t_0_).

Whereas in NDSC the spectral variations are clearly visible within 40 minutes, in NDRC the spectra show much reduced spectral variations in the same period of time (Fig. 6). After 10 minutes from the culture medium removal, the signal intensity at 1580 cm^−1^ transiently decreases (upper panel of Fig. 6c), in fact: after 20 minutes this band recovers the initial intensity(Fig. 6b) and remains stable until the end of the series(Fig. 6d). At the same time, an increase of other signals, such as lipid (1450 cm^−1^), cyt c (1310 cm^−1^) and protein (1250 cm^−1^) bands, occurs (Fig. 6b). The intensity of these signals slightly decreases after 30 minutes and the differences with respect to the control in the 1300–1700 cm^−1^ range are much reduced (Fig. 6c). In this case, the recovery of 1580 cm^−1^ intensity suggests the occurrence of a reversible process, which is probably connected to the adaptation of the cell metabolism to starvation. From the morphologic point of view, the bright field images(Fig. 6a) shown a significant cell shrinkage after 10 minutes; after this initial variation the cell morphology recovers the original shape as well as the spectrum recovers the original profile with small differences at 1100–1300 cm^−1^.

### DMSO treatment of U251 cell line

We also studied the effect of the addition of dimethyl sulfoxide (DMSO). This solvent, which is frequently used in biological studies, quickly modifies the cell volume^37^ and induces apoptosis when present at a high concentration in several cell lines^38–40^. We studied the effect of 10% v/v DMSO addition since this concentration is typically employed in cryopreservation protocols^41^. Notable that in chronic use of DMSO the toxic concentration limit for *in vitro* studies is about 1% v/v, above which it causes apoptosis due to the plasmatic membrane pore formation^42–44^. U251 cells were washed three times with PSS and then incubated at ambient temperature for 10 minutes with a solution of DMSO/PSS 10% v/v. After treatment, the DMSO solution was washed out and the sample was analysed in physiological solution by Raman microspectroscopy.

**Figure 7:**
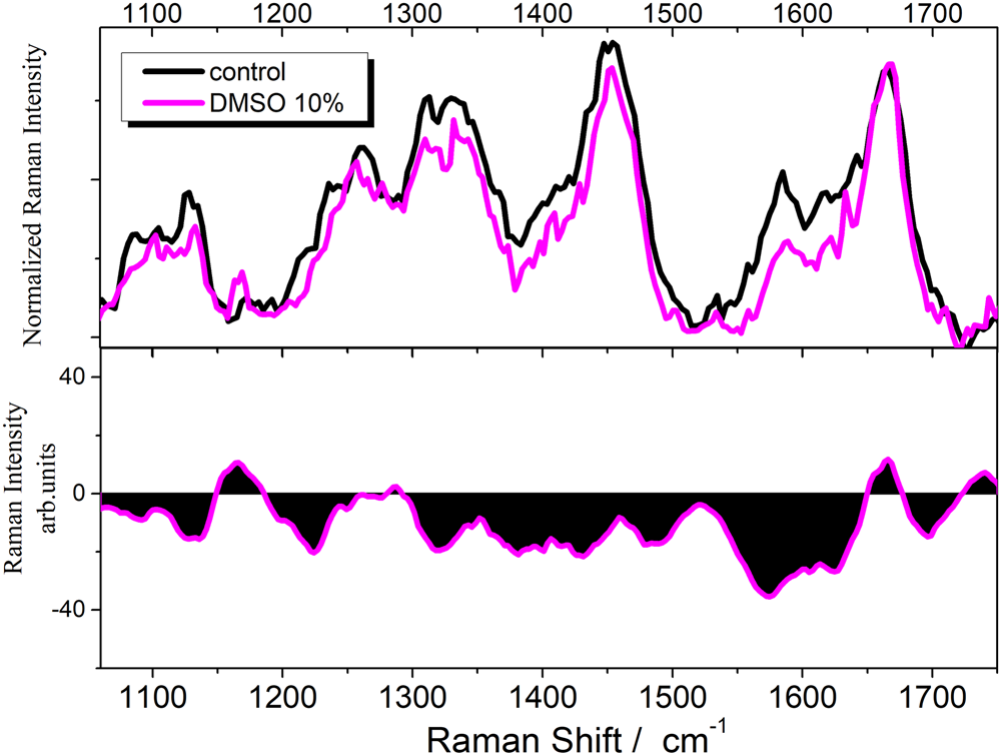
Raman spectrum of DMSO-treated cells. Comparison between the spectrum of U251 cells treated with a solution of DMSO 10% v/v (magenta line) and the non-treated control (black line): on lower panel the difference spectrum obtained by subtracting the control to the spectrum of DMSO treated cells is shown.

In Fig. 7, the average spectrum of U251 cells treated with a solution of DMSO 10% v/v is compared with that of the non-treated control in the 1060–1760 cm^−1^ range.

The spectrum of the treated sample is characterized by a lower intensity of various spectral features compared with the control. The difference spectrum on the lower panel of Fig. 7 shows negative contributions in the 1200–1650 cm^−1^ region and indicates a maximum variation at 1580 cm^−1^.

**Figure 8:**
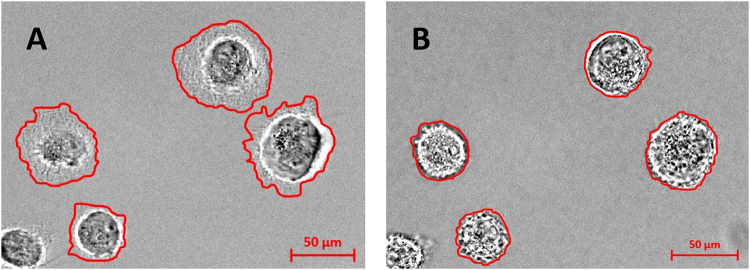
Optical images of DMSO-treated cells. Magnification image of U251 cells before (**A**) and after wash-out (**B**) from 10% of DMSO treatments (10 min). Red lines indicate projection area of the cells.

The microscope images displayed in Fig. 8 suggest a change of cell volume. It is generally observed that DMSO washout with physiological solution causes cell swelling which is followed by regulatory volume decrease (RVD); RVD needs several tens of minutes to reestablish the original cell volume^29,37^. In agreement with an increase of cell volume, we observed a lower intensity on proteins, nucleic acid and cyt c bands that is qualitatively and quantitatively different from the effects produced by H_2_O_2_ treatment (see Figs 3b and 7). Given the ubiquitous use of DMSO in many biological assays, the work has led to relevant results, which should be further deeply investigated.

## Discussion

In the recent years the number of studies that employ microRaman spectroscopy in the biomedical field is increasing. This powerful technique gained a lot of interest especially in the analysis of the neuronal system, going from the molecular investigation of the maturation process of neural model system^45^, to the monitoring of the induced differentiation process with retinoic acid of living neuroblastoma cells^46^. With regards to GBM, several efforts have been made to discriminate vital and necrotic glioblastoma tissues in order to test a possible *in vivo* Raman analysis for real-time intraoperative brain biopsy guidance^47,48^. Cancer tissues are often difficult to visually distinguish from normal ones and this has serious implications on the possibility to have a successful surgery or efficient targeted radiation- or chemo-treatments. Recently, Jermyn *et al*.^49^ tested an intraoperative Raman-based probe technique to detect invasive brain cancer in patients. Despite the important results so far achieved, the spectroscopic studies of single living GBM cells are still very limited.

In this regard, we studied Raman signal in U251 glioblastoma during hydrogen peroxide induced apoptosis^50^. In the apoptotic cells the cyt c, lipid, nucleic acid and protein signals have a reduced Raman intensity. The fragmentation of the nucleus starts at the beginning of the apoptotic process and can be the reason for the decrease of the nucleic acid bands in the Raman spectrum. Besides, in the Raman spectra of A549 cells, the action of Etoposide, which is known to initiate the death event in apoptotic cells, was related to the decrease of DNA, RNA and protein bands^51^. It was also suggested that the progress of internucleosomal DNA cleavage may induce the decrease of nucleic acid bands^52^. In our spectra the protein signals at 1250 cm^−1^ decreases in intensity as well; this lower peak was also recorded during the apoptotic process^18,51^. In the spectrum of H_2_O_2_-treated samples, the decrease of 1450 cm^−1^ intensity is probably due to the loss of lipid molecule as apoptotic bodies in the extracellular environment. However, at an early stage of apoptosis the lipid content does not change and lipids are not subjected to enzymatic degradation.

We also studied the Raman signal in cells during acute (within 40 minutes) nutrient depletion. In accordance to heterogeneous glioblastoma subpopulation composition, we observed two types of responses: one cell subpopulation (NDSC) displays an overall decrease of Raman signals (nucleic acid an protein) and cell swelling behaviour, and the second subpopulation (NDRC) displays a transient overall increase of Raman signals associated to cell shrinkage. Cell volume variations in both subpopulations were evaluated by estimation the projection area of cells by using microscopy analysis. We hypothesize that the Raman signal variations observed at short time (40 minutes) is not associated to a variation of macromolecule composition, as observed after 4 hours following hydrogen peroxide treatment, but to a concentration increase or decrease of intracellular macromolecules as a consequence of cell volume changes. Also DMSO treatment was demonstrated to promote volume variations and particularly cell swelling within few minutes after wash-out^37^: this induced a spectral change similar to the one observed for NDSC subpopulation.

Possible mechanism explaining how nutrient depletion induces swelling could be related to the reduced activity of the Na/K pump as a result of the block of glycolytic metabolism and consequent intracellular ATP decrease. The impair of Na/K pump decreases the sodium extrusion that is crucial to compensate the ionic distribution of Gibbs-Donann effect^53^. The dependence of glucose in glioblastoma cell for the production of ATP, also in the presence of oxygen, is widely reported in GBM cancer and known as Warburg’s effect^54^. We also observed a subpopulation that displays a transitory cell shrinkage during nutrient depletion as possible consequences of intracellular osmolyte efflux and activation of regulatory volume increase (RVI) or other mechanisms needed to compensate the volume variation. As possible mechanism for a transitory volume decrease is the transient activation of potassium and chloride currents, which promotes KCl efflux from the cell. Swelling activated chloride currents (IClsw) are central to the volume regulation process in glioblastoma cells^55^, and their activity is increased by cell swelling and blocked by cell shrinkage. It is possible that the basal activity of IClsw observed in glioblastoma^56^ initially promotes chloride efflux and cell shrinkage that, in turn, close the IClsw activity. The resulting activation of RVI^21^, or intracellular sodium accumulation as a consequence of Na/K pump impairment resulting from intracellular ATP depletion, restores the initial cell volume. The dependence of cell swelling on both Na/K pump impairment and cell shrinkage by IClsw activation are currently under investigation in our laboratory.

Our results demonstrate the sensitivity of Raman microspectroscopy to detect rapid (within few minutes) variations of macromolecule concentration due to oxidative stress and cell volume changes. An important aspect to consider is the experimental setup and the kind of substrate where cells were grown. The health of the cell may be affected by the interaction with the substrate^25,57^. In fact, the receptors regulating adhesion are involved in signal transduction, and the functions connected with the process are all governed, at least partially, by cell adhesive interactions^57^. Since the properties of biomaterials affect the cell membrane interaction with the surface, the choice of the substrate for the cell culture is crucial. The use of silicon in miniaturized mechanical and electronic devices is widespread due to its properties as a semiconducting and high-precision mechanical material. Moreover, Si is characterized by high biocompatibility and due to proper integration of electronics and biological systems, it is widely used in biomedical applications in the field of neurosciences. For instance, it is used in functional electrode stimulation^58^, Parkinson’s disease^59^ and electrode-neuron implants^60^. New trend in recent decade is towards lab-on-a-chip applications for live cell Raman imaging^61^. We emphasize that Raman analysis of cells grown on Si substrate represents a valid method to study cell processes such as apoptosis and cell volume regulation.

## Methods

### Cell culture and treatments

U-251 cell line^62^ was purchase from Cell Lines Service (CLS), GmbH Culture Collection (Eppelheim, Germany) and used at passages 50–70. Cells were grown in Dulbecco Modified Eagle’s Medium (DMEM) containing 10% (v/v) heat-inactivated fetal bovine serum (FBS), 2 mM L-glutamine, 100 U/mL penicillin, 100 U/mL streptomycin (SIGMA Aldrich, St. Louis). The cells were cultured at 37 °C in a humidified atmosphere with 5% CO_2_ and passaged as needed using 0.25% trypsin-EDTA. When cells reached 80% confluence, they were trypsinized and seeded at a density of 1 × 10^5^ cells/cm^2^ for 24 h onto the silicon supports sterilized either by ethanol immersion and by ultraviolet (UV) irradiation.

Cell apoptosis was induced by 4 hour treatment with 300 mM H_2_O_2_ in DMEM solution. The DMSO-induce cell swelling was obtained by 10 minute exposure to 10% v/v of DMSO followed by washout with physiological Saline Solution (PSS) containing 106.5 mM NaCl, 5 mM KCl, 2 mM CaCl_2_, 2 mM MgCl_2,_ 5 mM MOPS, 20 mM D-(+)-Glucose, 30 mM Sodium Gluconate (pH 7.25)^55^. Measurements was executed within 10 minutes from DMSO removal. Nutrients depletion treatment was obtained by changing the medium solution with a 1X Phosphate Buffer Saline (PBS) solution w/o calcium and magnesium (Euroclone, ECM4004XL). All experiments were performed at room temperature.

### Raman microspectroscopy

Raman spectra were collected using a micro-Raman setup equipped with a solid state laser at λ = 532 nm whose power is reduced to less than 10 mW to avoid photo-induced damages of the cells during the acquisition time. A back scattering geometry was realized using the 50x long working distance objective of an OLYMPUS microscope MOD BX40. The microscope has a digital camera, and it is attached to a computer. The scattered radiation was analysed by an iHR320 imaging spectrometer Horiba Jobin- Yvon. The number of scans was three in case of nutrient depletion in order to reduce the acquisition time and follow the time evolution of spectral variations; ten scans were averaged under H_2_O_2_ and DMSO treatments. The scans were accumulated within 30 s integration time using a 5 cm^−1^ resolution.

To perform Raman measurements the culture medium was removed and the cells were washed three times with PSS. The culture medium, indeed, causes an intense background on Raman spectrum that interferes with the cell signals. To exam the cell response to each stressing condition (H_2_O_2_ addition, nutrient depletion and DMSO addition), at last ten different cells were analysed for a single culture and the experiment was performed in triplicate to test the reproducibility of results. For each cell, we performed single point analysis with a 1.3 μm spatial resolution in correspondence of the nucleus. The Raman spectra shown above were obtained from the average of ten-fifteen different acquisitions.

The spectra were normalized to the OH-stretching vibrational modes, and the water contribution was subtracted from the spectrum of the cellular sample; the water spectral profile was measured for each sample on the same substrate (in a region free from cell adhesion). Spectra were also corrected by using a multipoint baseline procedure and then normalized to the intensity of the 1660 cm^−1^ band. This band was used as a reference since we observed that it is a less efficient probe of the fast cell response to external stimuli, in fact: after subtraction of water spectrum we observed only small intensity variations at 1660 cm^−1^ from sample to sample. On the contrary, different spectral profiles were observed at lower frequencies for treated samples. We normalized to the intensity of the high frequency side of amide I band because at 1600–1640 cm^−1^ the contribution of cytochrome ν_10_ band^63^ and of C=C stretching mode of lipids^64^ are not negligible at 532 nm excitation. The silicon Raman spectrum is silent only above 1060 cm^−1^, but the characteristic silicon Raman band are excessively intense to be effectively subtracted to the cell spectrum. For this reason, the spectra of the cell grown on silicon were not considered below 1060 cm^−1^. The effects of stressing treatments are also described by differences between spectra of treated samples and control; these difference spectra on Figs 3,5,6 and 7 are shown on the same y-scale to make a quantitative comparison among different experimental conditions.

### Acridine Orange Staining

Cells was washed twice with PBS, then incubated at 37 °C for 15 min with 1 µM of Acridine orange. After incubation, the samples were washed twice with PBS then visualized under Axio Examiner (Zeiss) florescence microscopy. Cells were illuminated with 488 nm excitation filter and a 550 nm emission/barrier filter whereas lysosomes were illuminated using an excitation filter of 550 nm and a long pass 610 nm emission/barrier filter.
